# How Important are Cereals and Cereal Products in the Average Polish Diet?

**DOI:** 10.3390/nu11030679

**Published:** 2019-03-21

**Authors:** Wacław Laskowski, Hanna Górska-Warsewicz, Krystyna Rejman, Maksymilian Czeczotko, Justyna Zwolińska

**Affiliations:** Department of Organization and Consumption Economics, Faculty of Human Nutrition and Consumer Sciences, Warsaw University of Life Sciences, 02-787 Warsaw, Poland; waclaw_laskowski@sggw.pl (W.L.); krystyna_rejman@sggw.pl (K.R.); makysmilian_czeczotko@sggw.pl (M.C.); justyn.zwolinska@gmail.com (J.Z.)

**Keywords:** cereals, cereal products, energy intake, nutrient intake, food sources, household budget survey

## Abstract

The main aim of this study was to identify the food sources of energy and 28 nutrients from cereals and cereal products in the average Polish diet based on data from a nationally representative sample of the Polish population attending in 2016 Household Budget Survey (i.e., 36,886 households). The contribution of energy and nutrients from cereals and cereal products were compared with reference values. The detailded analysis included five main groups and nine sub-groups of cereal food category. Our findings indicated that cereals and cereal products contributed 30.4% of total dietary energy supply, providing a significant percentage of six nutrients to the average Polish diet (i.e., 64.1% of manganese, 51% of carbohydrates, 48.5% of dietary fibre, 34.1% of iron, 33.6% of folate, and 31.3% of copper). Supply at the level of 20–30% was observed for protein, thiamin, phosphorus and zinc, and at 10–20% for polyunsaturated fatty acids (PUFA), sodium, potassium, calcium, riboflavin, niacin, and vitamin B6. For other nutrients i.e., total fat, saturated fatty acids (SFA), monounsaturated fatty acids (MUFA), cholesterol, iodine, vitamins: A, D, B12, and C, the share of cereals and cereal products contribution was below 10%. Cereals and cereal products were the major food category in meeting the reference values for the Polish population in case of manganese, carbohydrates (approximately 100%), and sodium (50%). The reference values was reached at the level of 30–40% for dietary fibre, protein, iron, copper, zinc, phosphorus and thiamin, and 20–30% for energy, magnesium, folate, niacin, riboflavin, vitamins B6 and E. For such nutrients as total fat, SFA, and potassium, the fulfillment of the reference values amounted to 10–20%. Our results document the importance of cereals and cereal products in the Polish diet, which should be emphasized from a nutritional and health point of view.

## 1. Introduction

Cereals and cereal products are staple foods in most human diets [1,2,3], in both developed and developing countries, providing a major proportion of dietary energy and nutrients. They are composed of approximately 75% carbohydrates, mainly starches and about 6–15% protein, contributing in global terms more than 50% of energy supply [4]. The importance of cereals and cereal products is also supported by the fact that global food security depends to the greatest degree on cereal production, which yearly amounts to approximately 2600 million tons [5].

The role of cereals and cereal products are frequently analyzed in the scientific literature in relation to the level of consumption and the nutritional value [6,7,8]. Some cereal products (breads, rolls and tortillas, ready-to-eat cereals, quick breads and bread products) are described as contributors of folate, iron, thiamin, niacin, dietary fibre [6], manganese and zinc [9,10]. Consumption of whole grain cereal products is associated with higher diet quality and nutrient-dense foods delivering protein, lipids, B vitamins (including thiamin, niacin, riboflavin), vitamin E, and minerals (calcium, magnesium, potassium, phosphorus, iron, and sodium) [7]. The bioavailability of manganese should be emphasized [11] due to its role in metabolic processes [10,12,13,14,15,16,17] as well as the functioning of nervous, immunological and reproductive systems [14,15,16]. Many studies have described the negative effect of phytates, which are natural substance found in cereals and cereal products, on the bioavailability of minerals and trace elements. The interaction between phytic acid and minerals should be taken into consideration to ensure high bioavailability and adequate supply of them [11].

Currently, the importance of cereals in the diet, particularly wholegrain ones, is being explored due to the presence of dietary fibre and bioactive compounds. Dietary fibre components are unevenly distributed in a grain and their highest concentration occurs in the outer tissues [18]. Whole kernel or coarsely milled grains contain more dietary fibre and phytochemicals with potential anti-inflammatory and antioxidant properties than refined grains [19]. On the basis of a series of systematic reviews and meta-analyses, it can be stated that there is substantial epidemiologic evidence that dietary fibre and wholegrain foods are associated with decreased risk of diet-related non-communicable diseases (DRNCD) [20,21,22,23,24]. Diet with high levels of dietary fibre and whole grains result in reduced risk of all-cause and cardiovascular related mortality [21,22,23,25,26,27,28], atherosclerotic cardiovascular diseases [21,23,29,30,31,32,33], ischemic stroke [27,32,34], type 2 diabetes [21,23,30,33,35,36], obesity [29], and some types of cancers [21,23,27,30,33,37,38]. Dietary fibre and resistant starch provide substrate for colonic microbial fermentation, leading to the production of short chain fatty acids that are a direct energy source for the colonic epithelium and influence hepatic insulin sensitivity [18]. The importance of dietary fibre in the prevention of colorectal cancer remains controversial. However, studies conducted on large population samples showed that high intakes of dietary fibre, especially from grains, cereals, and whole grain cereal foods are associated with reduced risks of colorectal [23,28,39,40,41,42] and gastric cancer [33,41]. Many other studies strengthen the evidence for dietary fibre protective role in the prevention of colon and/or rectum cancer [43,44,45,46,47]. An inverse association has been observed between consumption of whole grains and cereal fibre and a reduction of total and cause-specific mortality [25]. However, according to the newest World Cancer Research Fund International report the role of whole grains consumption in reducing the risk of colorectal cancer is not yet convincing. However, scientific findings have allowed the statement that whole grains probably decrease the risk of these types of cancer [48]. 

The importance of cereals, especially whole grains, in the prevention of the DRNCD is especially important due to the three stages of the nutrition transition process which are revealed in changing dietary patterns and lifestyle resulting from rapid economic growth [4,49]. The first stage has occurred in both developed and developing countries and is known as the “expansion” effect [50] or “receding famine” with higher consumption of cheaper food of vegetable origin: starchy, low fat, high dietary fibre and low variety [49]. This is accompanied by improvements in food availability and the significant rise in dietary energy supply observed globally and the gradual elimination of dietary deficiencies, thus improving the overall nutritional status of the population [4]. The second stage (“substitution effect”) results in a shift in the structure of food consumption with no major change in the overall energy supply. This shift is primarily from carbohydrate-rich staples to vegetable oils, animal products and sugar [51]. In the second stage DRNCDs predominate, as a result of an energy-dense diets that are high in total fat, cholesterol, sugar and other refined carbohydrates, and low in polyunsaturated fatty acids and dietary fibre, that are often accompanied by an increasingly sedentary lifestyle [49]. Rapid urbanization has become the main driving force behind this qualitative nutrition transition stage, followed by technological changes, facilitating work and leisure, food processing development, mass media growth [49,52], food marketing, and the policies of trade liberalization [51]. The prevalence of diet-related diseases together with global population growth and the imbalance of ecosystems determine the shift to the next stage of nutrition transition. To change diet to being more sustainable, which are profitable both for the people and the planet is the immediate challenge. The EAT-Lancet Commission, a multinational initiative launched for food system transformation, describes an universal healthy reference diet based on sustainable consumption rules. This diet focuses on an increase in consumption of healthy foods, such as vegetables, fruits, whole grains, legumes, and nuts, and a decrease in consumption of unhealthy foods, such as red meat, sugar, and refined grains that would provide major health benefits [53].

In terms of the importance of cereals and changes in food consumption patterns, it should be highlighted that the analysis of energy and nutrient sources is crucial to assure the adequate nutritional quality of diets. The aim of this study was to identify food sources of energy and 28 nutrients from cereals and cereal products based on data from a representative sample of the Polish population drawn by the Central Statistical Office for the purpose of Household Budget Survey in 2016. The results of the contribution of energy and nutrients were compared with reference values for the Polish population. 

## 2. Methods

### 2.1. Study Overview

Sources of energy and nutrient supply from cereals and cereal products were studied. The analysis covers energy and 28 nutrients: carbohydrates, dietary fibre, protein, total fat, saturated fatty acids (SFA), monounsaturated fatty acids (MUFA), polyunsaturated fatty acids (PUFA), cholesterol, calcium, phosphorus, sodium, potassium, magnesium, iron, zinc, copper, manganese, iodine, thiamin, riboflavin, niacin, folate, and vitamins B12, B6, A, D, E, and C. 

**Figure 1:**
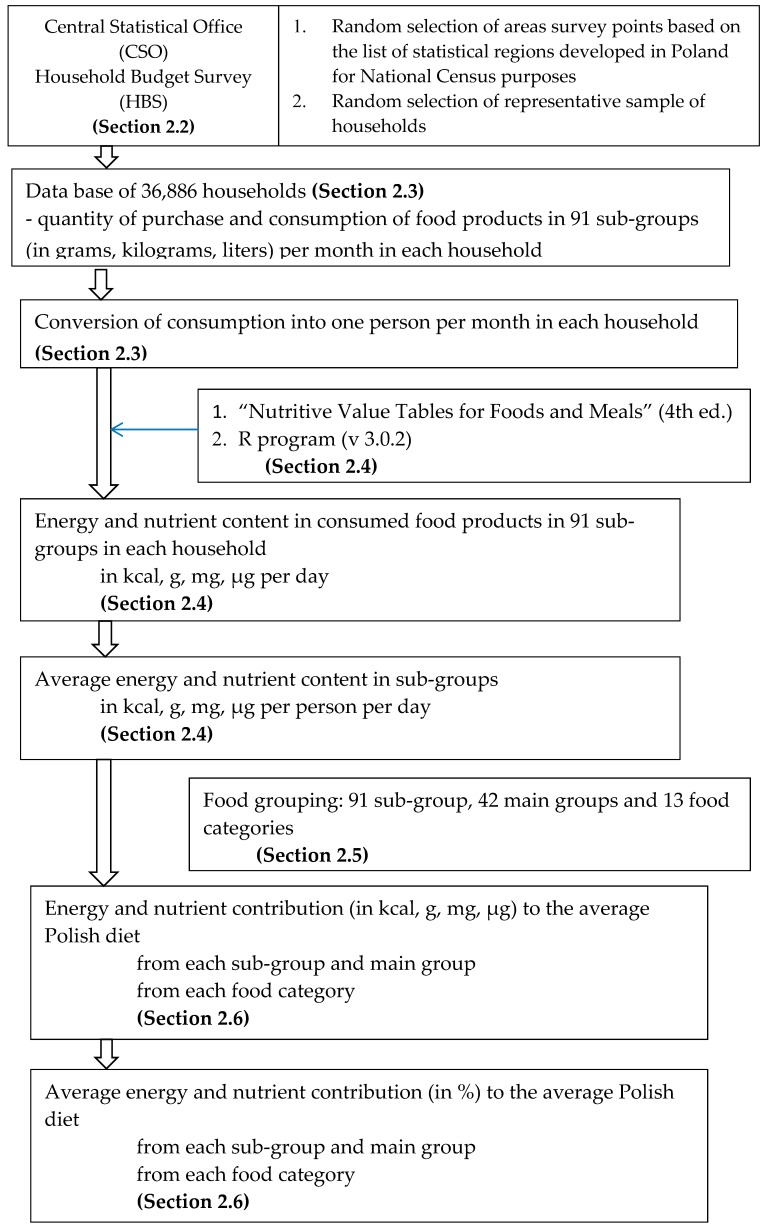
Research process.

The research process is presented in Figure 1 and described in Section 2.2, Section 2.3, Section 2.4, Section 2.5 and Section 2.6. 

### 2.2. Sample Selection Method

The Household Budget Survey (HBS), a representative method of data collection in Poland is organized and conducted by the Central Statistical Office, Social Surveys and Living Conditions Statistics Department in cooperation with the Regional Statistical Office in Łódź. In 2016, 36,886 households (total number of persons *n* = 99,230) participated in the HBS [54]. Each household kept records of expenditures, quantitative consumption and revenues in a special budget diary (paper booklet or electronic version available through Internet browser) for one month [54,55]. Surveys of households are conducted using a representative method based on a random sample, which gives the opportunity to generalize the obtained results for all households in the country. Since 1993, the HBS has been conducted using a total monthly rotation in a quarterly cycle. The "quarterly cycle" involves an additional interview conducted with households surveyed at the end of the quarter [54,55]. Additionally, in the case of non-response, the methods of a random replacement of households and sequential sampling were applied. For this purpose, the Central Statistical Office (CSO) uses two lists of households (first selection and reserve) to achieve the planned sample size and representativeness [55]. Detailed information related to sample selection process was published in our previous studies [56,57].

The data on each household participating in the survey are based on the “Household Budget Diary” and the questionnaire “Household’s Statistical Sheet”. The household budget survey is conducted by interviewers who are employees of statistical offices in voivodships (administrative regions of Poland; in EU statistics they correspond to NUTS2 type units). Their main tasks are as follows [54]:

- to visit every household at least four times a month, give instructions how to collect data and explain doubts;
- to conduct an interview in every household at the end of the month on income, expenses and living conditions;
- to collect, control and analyze the data recorded by the household;
- to enter data into the CSO system;
- to conduct interviews in quarterly cycle regarding household equipment, fixed assets and real estate.

### 2.3. Food Consumption Data

The basis for the calculations was the detailed data from each of the 36,886 households with information characterizing households (i.e., location, income, education level, etc.) and data on purchased and consumed products in terms of quantity (in grams, kilograms, liters) and value (in Polish zloty). Food grouping was presented in our previous article [57] and is included in Supplemental Table S1. Data on the consumption were converted into one person per month, using the number of persons in the household and the number of days of in-home nutrition. According to the CSO methodology, the moment of consumption of food products is the moment of their acquisition by a household. For each of the 91 sub-groups we have received the amount of consumption expressed in grams, kilograms or liters per 1 person per one month and day. These data were the basis to determine the nutritional density of the average Polish diet [58], and to identify the food sources of nutrients [56,57].

In this study we analyzed cereals and cereal products as a source of energy and 28 nutrients. 

### 2.4. Energy and Nutrient Content—Statistical Calculations and Method

To convert the amount of consumption of a particular food product expressed in grams, kilograms and liters into energy (in kcal) and nutrients content (in mg, µg) we used:

1. “Nutritive Value Tables for Foods and Meals” (4th ed.) [59]—the official in Poland food composition tables;
2. R program (v 3.0.2, The R Foundation for Statistical Computing, Vienna, Austria), system and environment for statistical computation [60,61,62].

Such a conversion was made for each of sub-groups in two stages: first for each household, and then as an average value per statistical person in each household. 

The ”Nutritive Value Tables for Foods and Meals” [59] were developed and updated in 2017 by the National Food and Nutrition Institute. From the database of 1100 products and assortment items, we used 930 products. The average energy and nutrient content were calculated considering, if necessary, the weights resulting from the known or estimated proportion of the consumption of the product relative to the others in the group [58].

The R environment is a computer language and an integrated software facility for data calculation, implementation of statistical techniques and graphical display [60,61,62]. It allows the integration of the HBS database and data from “Nutritive Value Tables for Foods and Meals” to calculate the average intake of energy and nutrients using special writing functions. The weight of corrections was implemented to improve the representativeness of the results and account for the size of the household. This allowed us to consider the results as being representative for the population of Poland [58,63].

In addition, energy and nutrient supply were compared to the reference values. For this purpose, we used the reference values for energy and nutrients for the Polish population, developed and published by the National Food and Nutrition Institute [64]. Results were presented as a percentage of fulfillment of the reference values for energy and 28 nutrients.

### 2.5. Food Grouping

Food products were aggregated into 91 sub-groups, 42 main groups and 13 categories (Table S1). The classification has been modified and published previously [54,65,66,67,68]. For this study the food category “cereals and cereal products” was broken into five main and nine sub-groups as shown in Table 1. 

### 2.6. Results Format

For the purpose of this study, the level of supply of energy and 28 nutrients were calculated for one cereal food category; five main groups; and nine detailed sub-groups. 

The mean nutrient intake from these foods was expressed as a percentage of the total dietary intake of the analyzed nutrient and ranked in descending order. A three-stage method of presenting the results was adopted:

(1) the amount of consumption of cereals and cereal products—Section 3.1
(2) the amount of intake, the share of main groups from the cereal category in terms of their contribution to energy and 28 nutrients intake, and the share (%) in fulfillment of reference values—Section 3.2 (energy), Section 3.3 (macronutrients), Section 3.4 (minerals), and Section 3.5 (vitamins)
(3) share of five main groups and nine sub-groups from the cereal category in terms of their contribution to intake of energy and 28 nutrients – Supplemental Tables S1–S30.

## 3. Results

**Figure 2:**
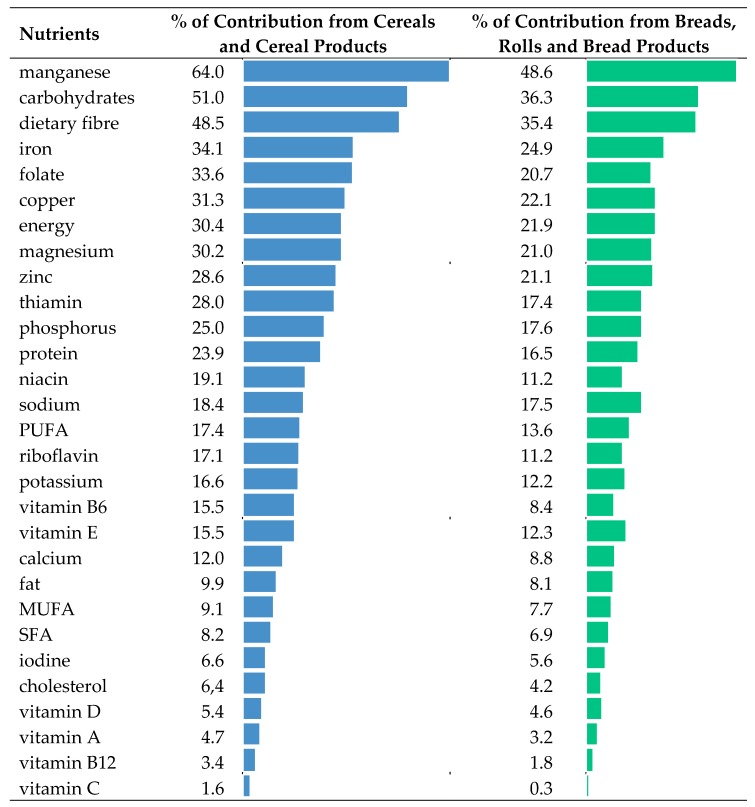
Contribution of energy and nutrients in the average Polish diet from the category of cereals and cereal products and from breads, rolls, and bread products.

Average consumption of cereal products is presented in Table 2. Food sources of energy and 28 nutrients from cereals and cereal products are shown in Table 3, Table 4, Table 5, Table 6 and Table 7 and Figure 2, as well as in the Supplemental Tables S2–S30.

### 3.1. Cereal and Cereal Product Consumption

Consumption of cereals and cereal products is presented in Table 2. The consumption of these products is dominated by bread and rolls with a quantity of almost 130 grams per person per day. Second place in the ranking was taken by products which are substitutes for fresh bread (quick bread and bread products). It is worth noting that wheat flour turned out to be the third group in terms of amount consumed. This indicates that the preparation of traditional flour dishes (such as pancakes, macaroni, many types of dumplings, gnocchi, crumpets etc.), sweet bakes (cakes, pies, cupcakes, torts, etc.) as well as bread is still practiced in Polish households, especially in small cities and rural areas [69]. 

### 3.2. Cereals and Cereal Products as Sources of Energy

Cereals and cereal products provided 30.4% of energy supply in the average Polish diet (i.e., 687 of 2261 kcal/day). This means fulfilling of reference value for energy at 27.7%. Table 3 shows that bread, rolls and other bread products were by far the largest contributor among the main cereal food groups. Second and third place in the daily supply of energy from cereals were took flour, bran, cooking ingredients and then pizza, pasta and other flour dishes respectively. The top three sub-groups were: bread and rolls (16.4%), quick breads and other bread products (5.5%), and wheat flour (3.3%) (Table S2).

### 3.3. Cereals and Cereal Products as Sources of Carbohydrates, Dietary Fibre, Protein, Total Fats, Fatty Acids, and Cholesterol

As can be seen in Table 4, cereals and cereal products provided approximately half the average supply of carbohydrates and dietary fibre, one fourth of the protein and one sixth of the polyunsaturated fatty acids, while less than one tenth of the saturated and monounsaturated fatty acids and cholesterol came from the cereal category. The highest level of fulfilling the reference values was observed for carbohydrates, and then for dietary fibre and protein. Bread, rolls and other bread products were the top contributors, while the two next ranked main groups supplied considerably less. Among the sub-groups, bread and rolls, as well as quick breads and other bread products were the top sources of carbohydrates, protein and poly-unsaturated fatty acids as shown in the Supplementary Tables S3–S10.

### 3.4. Cereals and Cereal Products as Sources of Minerals

The largest share of cereals and cereal products in the supply of minerals was recorded in the case of manganese (over 60%), followed by iron and copper (Table 5). For zinc and phosphorus, the share of cereals and cereal products amounted to 20–30%, while for sodium, potassium and calcium this was 10–20%. The highest supply compared to the reference values was observed for manganese and sodium. For phosphorus, copper, iron, and zinc the reference values were reached at the level of 30–40%. Bread, rolls and other bread products were ranked first among all analyzed product groups. The second and third positions included various product groups, and the details are presented in the annex in the Tables S11–S20. 

### 3.5. Cereals and Cereal Products as Sources of Vitamins

As shown in Table 6, cereals and cereal products provided one third of the total supply of folic acid. A significant share of 28% was recorded for thiamine. For other B vitamins (niacin, riboflavin, vitamin B6) and for vitamin E, cereals and cereal products contributed 15–20% of average daily supply. In the case of vitamins A, D, C and B12, cereals and cereal products were rather unimportant sources of them, providing up to 5% of the average daily intake. The highest fulfillment of the reference values was observed for thiamin (above 30%), then for folate, niacin, riboflavin, vitamin B6, and E (20–30%). The reference value for other vitamins was covered below 10%. For most of the analyzed vitamins, the sub-groups containing bread, rolls and bread products were the main food source, with the highest proportion of folate. The second sub-group was flour, bran, and cooking ingredients for folate, thiamin, niacin, riboflavin and vitamin B6, as well as pizza, pasta and other flour dishes for vitamins: E, A, D, B12, and C. Detailed information about the sources of all nutrients are presented in Supplemental Tables S21–S30.

### 3.6. Result Summary

In conclusion, our results emphasize the importance of cereals and cereal products in providing nutrients at a higher level than energy for manganese, carbohydrates, dietary fibre, iron, folate, and copper (Figure 2). The main group from cereal category with the largest share in the supply of almost all of the analyzed nutrients and energy turned out to be bread, rolls, and bread products (Figure 2, Table 7).

## 4. Discussion

Cereals and cereal products form a significant part of the diet in Poland as contributors of energy and many nutrients. Our findings based on the 2016 HBS indicated that this food category contributed 687 kcal, which means 30.4% of total energy supply in the average Polish diet and almost 28% in comparison to the energy reference value for the Polish population. Cereals contribution to dietary energy intake is widely diversified between regions and countries. In developing Asian countries with rice-based diets, cereals contribute as much as 70–80% of energy intake; while, in high income countries with predominantly livestock-based diets cereals provide only 20–30% of total dietary calories [70]. In Europe, the share of cereals and cereal products in terms of energy contribution to the average diet amounts to 40% [71]. The structure of the average Polish diet reflects the second stage of the nutrition transition process, which is revealed in changing dietary patterns and lifestyle. This is the effect of transformation processes that started in Poland at the beginning of the 1990s and resulted in dynamic changes in the food market, evolving consumption patterns and social inequalities. According to the yearly CSO aggregated results of the Polish household budget surveys, the consumption of cereals and cereal products decreased by 23.6% during the last 10 years [54,72]. 

Findings from our research related to the importance of main groups and sub-groups within cereal category were compared to data from surveys conducted among American [6,7,8,66], British [73,74], Spanish [75], Japanese [10] and the Canary Islands’ [9] population. In the average Polish diet, the highest share of energy contribution was related to bread, rolls and bread products (21.9%). In the American population, the highest ranked food products were yeast breads and rolls (7.2%); and cakes, cookies, quick bread, pastry and pies (7.2%) [66]. In the UK diet, where cereals and cereal products contributed 34.5% of total energy supply, bread also was the main source of energy, however contributing almost two times less than in Poland (11.8%) with a predominant share of white bread (7.3%) [74]. 

Differences in the shares of particular cereal products in providing energy to the average Polish, US and UK diets [6,7,8,66,73,74] result from the different consumption structure of this food category. In Poland, the consumption of bread and rolls, despite the declining tendency is at a higher level [76] compared to other countries [77,78]. Data from the CSO surveys in Poland indicate that in 1998 monthly consumption of bread amounted to 7.5 kg per person in a household, while in 2007 reached only 5.3 kg [79,80], 3.9 kg in 2014 [69], 3.5 kg in 2016 and 3.3 kg in 2017 [81]. It means that, during the last 20 years, the consumption of bread in Poland decreased by 56%. The decline in bread consumption is the result of an increase in average income and standard of living improvements. It is known in the economics of consumption that staple food consumption, e.g. bread, is characterized by a negative income elasticity of demand [79]. A decrease in bread consumption is related to overall changes in eating patterns in Poland as well as an increase in the supply and consumption of bread substitutes and convenience cereal products, including ready-to-eat cereals, fast foods [20] and quick breads [79]. At the same time, the decision-making process of bread purchasing is determined by sensory [20,82,83], health and nutrition attributes [20,82,83,84,85,86,87,88]. 

Of the analyzed macronutrients, cereals and cereal products are important sources of carbohydrates, dietary fibre and protein. In 2016, they delivered almost 140 g of carbohydrates, giving more than half of total daily intake and more than 100% as compared to the reference value. When considering main groups, bread, rolls, and bread products delivered 36.3% of carbohydrate supply. Findings for carbohydrate contribution to the American diet indicated in the first place yeast breads and rolls (10.9%), followed by cakes, cookies, quick bread, pastry, and pie (8.9%) [66]. Data for the British population showed that bread contributed 18% of carbohydrate intake (total percentage of this macronutrient from cereal category was 47%) with the highest share related to white bread (11.2%) [74]. 

Dietary fibre contribution to the average Polish diet from cereals and cereal products amounted to 8.5 g (almost 50% of total daily supply), which means covering the reference value at approx. 36%. Our findings indicated decreasing tendency in lower dietary fibre consumption considering the results of previous studies, which have shown the consumption of 24 g per person per day in 1996–2005 [89], and of 28 g in 2000–2009 [90]. Moreover the role of cereals and cereal products in dietary fibre supplying is diminished, as earlier these food categories contributed 12.1 and 11.6 g of dietary fibre, respectively. This fact should be considered unfavorable from the nutritional and health points of view due to the importance of dietary fibre in reducing risk and prevention of many diseases (as presented in the introduction) [3–28,30,33,35–38,43–48]. According to the WHO/FAO’s population nutrient intake goals the dietary fibre intake should exceed 25 g per day [4]. The results of the current meta-analysis demonstrated that the beneficial reduction of DRNCDs risk is associated with daily intake of dietary fibre between 25 g and 29 g [23]. The Scientific Advisory Committee on Nutrition (SACN) in UK recommends even higher intake of 30 g of dietary fibre per day for adults, while current intake in this population group reached barely 18 g per day. In UK, cereals and cereal products contributed close to 42% of this amount, and 19% was contributed by bread [74].

In the average Polish diet, cereals and cereal products contributed almost 24% of total protein supply (30% as compared to the reference value), with main contributors of bread and rolls (13.2%). The protein contribution from cereals in the average UK diet was at the same level as in Poland (25%), including white bread (6.5%, i.e., twice less than in Poland) [74]. 

Among the analyzed minerals provided by cereals and cereal products special attention should be paid to manganese, iron, magnesium, zinc, and copper. Our findings indicated that cereals and cereal products contributed 2 mg of manganese and this amount gives almost two thirds of the total intake of this mineral (64.1%) and more than 100% of the fulfillment of the reference value. The main suppliers were bread, rolls, and bread products. Findings from the Canary Islands’ research also highlighted the role of cereals in manganese supply. The results for this population indicated that cereals and bakery products provided 46.3% of manganese [9]. Different food consumption patterns observed in the Japan population determine the structure of manganese contribution. According to the data from Japanese research, white rice was the main source of this micronutrient (24–33% of the daily intake), while bread contributed less than 3% [10]. It should be noted that only a small percentage of manganese from the diet is absorbed [91,92], as the presence of higher amounts of calcium, iron, phosphorus, phytates and fiber inhibits this process [91,92,93,94]. Therefore, the availability of manganese as well as other minerals from cereals and cereal products, especially whole grain, is limited, as they are partly bound in solid complexes with phytic acids and fiber [11,95]. In the face of these limitations, cereals and cereal products cannot solely provide the reference value for manganese in the Polish diet, as it is shown in the study. The other sources of this mineral are vegetables, potatoes, fruit and tea [64,96]. Especially very common in Poland habitual drinking of tea infusions may significantly contribute to the meeting of the reference value for manganese [97,98,99]. However, manganese is an essential nutrient due to its role [10,12,13,14,15] in terms of the metabolism of carbohydrates, amino acids, and cholesterol [10,16,17] as well as many functions of different systems [14,15,16]. 

With respect to iron, magnesium, zinc, and copper, cereals and cereal products provided a similar percentage (28–34%) of these micronutrients as it was stated for energy contribution. However, their share in meeting of the reference values was more diversified and amounted to 24–40%. The most important main group in delivering these micronutrients was bread, rolls, and bread products with special attention given to bread and rolls. Findings from the American surveys indicated that ready-to-eat-cereals were the most important food group from the cereal category in contribution of iron (15–19% of total supply) and zinc (approx. 4%) [8,66]. In the average Japanese diet, rice turned out to be the first group in terms of supplying copper (23–30%) and zinc (20–25%) [10]. According to a Spanish study, cereal and grain products were the food groups with the highest contribution to total iron intake (approx. 27%) [100]. Findings from the Canary Islands’ research noted that cereals contributed 19–21% of iron, copper and zinc to the average diets, while the share of bakery products related to these micronutrients contribution was significantly lower [9]. 

The data regarding the British population indicated a similar share of cereal category in supply of magnesium and zinc as in Poland (30 and 27%, respectively). Analogically to Poland, bread was the main source of these minerals in diet. It is worth noting that significant differences occurred in the supply of iron. In British population, on average, cereals provided almost 47% of this nutrient, and breakfast cereals (‘high fibre breakfast cereals’ and ‘other breakfast cereals’) was the major iron contributor to the diet (17%), which affected this discrepancy [74].

Differences in the importance of particular cereal product groups in providing these four minerals in the compared countries are the consequences of different dietary habits and consumption patterns. In the US about 16% of the population consumes ready-to-eat-cereals that are fortified with many nutrients and the consumers of these convenience cereal products had higher intake of shortfall nutrients [101] and more favorable nutrient intake profiles [102]. Moreover, the consumption of ready-to-eat-cereals was associated with higher quality and healthier diets [101,103]. This can explain the importance of ready-to-eat-cereals in providing iron in the average American diet. In Poland, fortified ready-to-eat-cereals are targeted at children and teenagers, while other population groups consume predominantly natural cereal flakes.

The role of zinc and iron should be emphasized when considering the nutritional value of vegetarian diets. Elimination of meat and meat products as a valuable source of macro- and micronutrients, particularly iron and zinc of high bioavailability [104,105,106,107] leads to lower absorption of these nutrients from vegetarian than from non-vegetarian diets [12,104,108,109]. It should be underlined that iron is required for many metabolic processes in the human body. Its deficiency is widely analyzed in the scientific literature [10,100,110,111,112,113,114]. The role of zinc is related to its structural role in proteins and cell membranes, and regulatory role in gene expression [10]. As far as copper is concerned, plant food is a good contributor of this micronutrient and vegetarian diets provide greater share of copper than non-vegetarian diets [12]. Copper is involved in energy and iron metabolism, and neurotransmitter synthesis and metabolism [10,17] as it is a nutrient protecting the human body against oxidative stress [13].

Phosphorus, sodium, potassium, and calcium were delivered by cereals and cereal products in a relatively low percentage (12–15%). The detailed data indicated that bread, rolls, and bread products were the main cereal products in contribution of these micronutrients. It is worth noting the unfavorable role of bread in supplying of sodium to the diet (18% of total sodium intake, which means half of the reference value). In case of the British population, the data is more unfavorable as cereal food category contributes 33% of sodium intake, including 17% from bread [74]. The addition of salt is of technological importance in the process of baking bread, but in developed countries excessive sodium intake is the main risk of hypertension [74]. According to the American survey, findings indicated lower contribution of phosphorus by yeast breads and rolls (4.5%); cakes, cookies, quick breads, pastry and pie (3.6%); and ready-to-eat cereals (2.3%) [66]. 

Cereals and cereal products are an important source of thiamin, folate, and in a smaller proportion of riboflavin, niacin, vitamins B6 and E. For folate, cereals and cereal products provided almost 34% of total supply (24% in comparison to the reference value). Many research showed shortages of folate in different population groups in Poland, and vegetables and grains were the main sources of this vitamin [115]. This is particularly important due to the function of this vitamin in the human body, especially in lowering the risk of NTDs (neural tube defects) [116]. In terms of the folate contribution, our findings indicated that the main food group was bread, rolls, and bread products (20.7%). Findings from the American research identified two main sources of folate: ready-to-eat cereals (18.7%), and yeast breads and rolls (16.6%) [66]. The main contributors of folate in the total diet of US adults ≥51 years old were ready-to-eat cereals (21.0%), and breads, rolls, and tortillas (13.4%) [8]. Again, the differences which occurred between Polish and US populations reflect diverse dietary habits in the case of ready-to-eat cereals consumption in combination with various approaches to food fortification in the public health policy.

Thiamin was delivered by cereals and cereal products in a similar percentage compared to the share of this food in dietary energy contribution. Our findings indicated the bread, rolls, and bread products (17.4%) as main contributors of thiamin. Data from the Spanish study indicated that cereals and grains contributed thiamin at a higher level (23.9%) [75]. In the American diet, the following food groups originating from cereals were identified in case of thiamin: yeast breads and rolls (14.7%), and ready-to-eat cereal (9.9%) [66].

This study has thoroughly documented the importance of cereals and cereal products in the Polish diet. Having in mind the urgent challenge to shift our diet to a more sustainable one, the Polish consumers should eat more whole grain foods to obtain more health benefits from the cereal category. The current dietary guidelines for the Polish population (the Pyramid of Healthy Eating and Physical Activity, updated in 2018) emphasize this recommendation as cereals and cereal products were placed at the second stage of the pyramid (after vegetables and fruit) and the accompanying tip encourage people to ‘eat cereal products, especially whole grains’ [117]. While dietary guidelines remain primarily health focused, synergies between health and sustainability mean that the guidelines include implicit sustainability messaging [118]. However, in many countries dietary guidelines were already updated and they combine tips regarding healthy food choices and the rules directly focusing on environmental or social aspects of sustainability.

The 2016 HBS sample size, consistent approach to classifying food products, and the use of the HBS methodology to record purchased and consumed food in terms of quantity and value are strengths of the presented study. Surveys of households are conducted using a representative method based on a random sample, which gives the opportunity to generalize the obtained results for all households in the country. The HBS analyzes consumption on a monthly basis; each household participates in the recording of purchased and consumed food for one month. However, there are some limitations, particularly the reliance on self-recording of information on consumption in a diary, which can lead to an under- and/or overestimation of consumption data, even though HBS uses well-established procedures to control all recordings. Additionally, the current edition of ”Nutritive Value Tables for Foods and Meals” (4th ed., 2017) includes new products and technological modifications, which may cause difficulties in the comparison of current results to data from earlier years. It should be noted that, in the CSO budget survey, households record the amount of all food obtained for the household, i.e., which was purchased, comes from self-supply or was received as a gift. The moment of consumption of food products is the moment of their acquisition by a household. In the case of non-response of households, the methods of a random replacement of households and sequential sampling are applied. The method of conducting a household budget survey also constitutes a difficulty in comparing with other studies on food sources of energy and nutrients. However, these limitations of survey methods are common and typical. Nevertheless, household budget surveys are the only representative method of data collection regarding food consumption and other living conditions of the Polish population. 

## 5. Conclusions

In conclusion, this study indicated that cereals and cereal products are important sources of energy and nutrients in the average Polish diet. The highest levels of contribution (above 30%) were observed for manganese, carbohydrates, dietary fibre, iron, folate, and copper. Cereals and cereal products provided 20–30% of the average intake of protein, thiamin, phosphorus and zinc, and 10–20% of the average supply of PUFA, sodium, potassium, calcium, riboflavin, niacin, and vitamin B6. Cereals and cereal products remain staple foods for the Polish population, as can be seen in the high contribution levels of these products to energy, carbohydrates, protein and some micronutrients. Our results document the importance of cereals and cereal products in the Polish diet from the nutritional and heath points of view. 

## Figures and Tables

**Table 1:** Main groups and sub-groups in the category of cereals and cereal products.

Main Groups	Sub-Groups
rice, cooked grains	rice
	groats and cereal grains
bread, rolls, bread products	bread and rolls
	quick breads and other bread products
pizza, pasta and other flour dishes	pasta, macaroni, noodles
	pizza and other flour dishes
flour, bran, cooking ingredients	wheat flour
	other flours
ready-to-eat cereals	breakfast cereals

**Table 2:** Average consumption of cereals and cereal products in Polish households.

Sub-Groups of Cereal Products	Average Consumption	
	g per Person per Month	g per Person per Day
bread and rolls	3 884	129.5
quick breads and other bread products	984	32.8
wheat flour	719	24.0
pasta, macaroni, noodles	420	14.0
rice	180	6.0
breakfast cereals	166	5.5
groats and cereal grains	142	4.7
pizza and other flour dishes	128	4.3
other flours	16	0.5

**Table 3:** Contribution of energy (%) from main cereal food groups and sub-groups in the average Polish diet.

Specification	Energy, %	Main Group Rank	Sub-Group Rank
Bread, rolls, bread products	21.9	1	
Bread and rolls	16.4		1
Quick breads and other bread products	5.5		2
Flour, bran, cooking ingredients	3.4	2	
Wheat flour	3.3		3
Other flours	0.1		9
Pizza, pasta and other flour dishes	2.6	3	
Pasta, macaroni, noodles	2.1		4
Pizza and other flour dishes	0.5		8
Rice, cooked grains	1.6	4	
Rice	0.9		6
Groats and cereal grains	0.7		7
Ready-to-eat cereal (breakfast cereals)	1.0	5	5
Total	30.4		

**Table 4:** Macronutrients, dietary fibre, fatty acids and cholesterol: contribution by cereals and cereal products to the average Polish, top three main groups and fulfillment of the reference values.

Nutrients	Average Polish Diet	Cereals and Cereal Products in		Bread, Rolls, Bread Products, %	Flour, Bran, Cooking Ingredients, %	Pizza, Pasta, Other Flour Dishes, %	Fulfillment of Reference Values, %
		g or mg	%				
Carbohydrates	270 g	137.8 g	51.0	36.3	5.9	4.2	103.9
Dietary fibre	17.6 g	8.5 g	48.5	35.4	4.4	(1)	35.9
Protein	77.9 g	18.6 g	23.9	16.5	3.1	2.5	30.0
PUFA	17.9 g	9.6 g	17.4	13.6	1.4	1.2	n.a.
Total fat	96.9 g	2.8 g	9.9	8.1	0.4	0.8	11.0
MUFA	37.4 g	3.4 g	9.1	7.7	(1)	0.8	n.a.
SFA	34.8 g	3.1 g	8.2	6.9	0.2	0.8	15.9%
Cholesterol	316 mg	20.2 mg	6.4	4.2	0	2.2	n.a.

(1) The third largest contributor were ready-to-eat cereals with 0.3% for MUFA and 3.2% for dietary fibre; n.a.: reference value not available.

**Table 5:** Minerals: contribution by cereals and cereal products to the average Polish diet, top two main groups and fulfillment of the reference values.

Nutrients	Average Polish Diet	Cereals and Cereal Products in		Bread, Rolls Bread Products, %	Flour, Bran, Cooking Ingredients, %	Fulfillment of Reference Values, %
		mg or µg	%			
manganese	3.1 mg	2.0 mg	64.0	48.6	(2)	103.5
iron	10.3 mg	3.5 mg	34.1	24.9	3.3	30.7
copper	1.1 mg	0.4 mg	31.3	22.1	2.6	39.0
magnesium	267.3 mg	80.8 mg	30.2	21.0	(2)	24.3
zinc	9.8 mg	2.8 mg	28.6	21.1	2.3	30.9
phosphorus	1160.2 mg	289.9 mg	25.0	17.6	2.1	39.3
sodium	3863.8 mg	710.6 mg	18.4	17.5	(1)	50.1
potassium	2617.9 mg	435.6 mg	16.6	12.2	1.4	12.9
calcium	644.1 mg	77.0 mg	12.0	8.8	0.8	6.5
iodine	154.6 µg	10.2 µg	6.6	5.6	0.3	6.3

(1) Second largest contributor were ready-to-eat cereals with 0.5%; (2) Second largest contributor was rice and cooked grains with 2.7% for magnesium and 4.5% for manganese.

**Table 6:** Vitamins: contribution by cereals and cereal products to the average Polish, their top two main groups and fulfillment of the reference values.

Nutrients	Average Polish Diet	Cereals and Cereal Products in		Bread, Rolls Bread Products, %	Flour, Bran, Cooking Ingredients, %	Pizza, Pasta and Other Flour Dishes, %	Fulfillment of Reference Values, %
		mg or µg	%				
folate	275.0 µg	92.3 µg	33.6	20.7	6.3		24.3
thiamin	1.3 mg	0.4 mg	28.0	17.4	4.5		31.9
niacin	16.2 mg	3.1 mg	19.1	11.2	3.8		20.7
riboflavin	1.6 mg	0.3 mg	17.1	11.2	2.8		23.1
vitamin B6	1.8 mg	0.3 mg	15.5	8.4	2.5		20.5
vitamin E	13.5mg	2.1 mg	15.5	12.3		1.0	20.0
vitamin D	4.6 µg	0.3 µg	5.4	4.6		0.8	1.5
vitamin A	1194.6 µg	56.3 µg	4.7	3.2		1.4	6.2
vitamin B12	4.5 µg	0.2 µg	3.4	1.8		0.8	5.8
vitamin C	91.4mg	1.4 mg	1.6	(1)		0.8	1.5

The table contains information only on two main sources of vitamins; empty places mean third and further positions of these sub-groups in the ranking; (1) pizza, pasta, and other flour dishes was the first contributor of vitamin C with 0.8% of supply.

**Table 7:** Rankings^1/^ of main cereal groups as contributors of energy and nutrients ^2/^ in the average Polish diet.

Food Group	energy	carbohydrates	dietary fibre	protein	total fat	SFA	MUFA	PUFA	cholesterol	calcium	phosphorus	sodium	potassium	magnesium	iron	zinc	copper	manganese	iodine	thiamin	riboflavin	niacin	vitamin B6	folate	vitamin B12	vitamin A	vitamin D	vitamin E	vitamin C
bread, rolls, bread products	1	1	1	1	1	1	1	1	1	1	1	1	1	1	1	1	1	1	1	1	1	1	1	1	1	1	1	1	3
flour, bran, cooking ingredients	2	2	2	2	3	3	4	2		3	2	5	3	4	2	2	2	4	5	2	2	2	2	2	3			3	
pizza, pasta and other flour dishes	3	3	5	3	2	2	2	3	2	2	3	3	2	5	4	3	4	5	2	5	3	5	5	3	2	2	2	2	1
ready-to-eat cereal	5	5	3	5	4	4	3	4		4	5	2	5	3	3	5	5	3	3	4	4	3	4	4	4	3		4	2
rice, cooked grains	4	4	4	4	5	5	5	5		5	4	4	4	2	5	4	3	2	4	3	5	4	3	5				5	

^1/^ the numbers for each nutrient denote the place of a given cereal main group in the ranking according to its contribution (%) to a given nutrient intake;^2/^ percentage of energy and nutrient contribution from individual main cereal groups: 
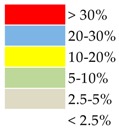
.

## Supplementary Materials

The following are available online at https://www.mdpi.com/2072-6643/11/3/679/s1 in Supplemental Section: **Table S1.** Food grouping for the purpose of the nutrient source analysis; **Table S2.** Food sources of energy contribution from cereals and cereal products in the average Polish diet; **Table S3.** Food sources of carbohydrates contribution from cereals and cereal products in the average Polish diet; **Table S4.** Food sources of protein contribution from cereals and cereal products in the average Polish diet; **Table S5.** Food sources of total fat contribution from cereals and cereal products in the average Polish diet; **Table S6.** Food sources of SFA contribution from cereals and cereal products in the average Polish diet; **Table S7.** Food sources of MUFA contribution from cereals and cereal products in the average Polish diet; **Table S8.** Food sources of PUFA contribution from cereals and cereal products in the average Polish diet; **Table S9.** Food sources of cholesterol contribution from cereals and cereal products in the average Polish diet; **Table S10.** Food sources of dietary fibre contribution from cereals and cereal products in the average Polish diet; **Table S11.** Food sources of calcium contribution from cereals and cereal products in the average Polish diet; **Table S12.** Food sources of phosphorus contribution from cereals and cereal products in the average Polish diet; **Table S13.** Food sources of sodium contribution from cereals and cereal products in the average Polish diet; **Table S14.** Food sources of potassium contribution from cereals and cereal products in the average Polish diet; **Table S15.** Food sources of magnesium contribution from cereals and cereal products in the average Polish diet; **Table S16.** Food sources of iron contribution from cereals and cereal products in the average Polish diet; **Table S17.** Food sources of zinc contribution from cereals and cereal products in the average Polish diet; **Table S18.** Food sources of copper contribution from cereals and cereal products in the average Polish diet; **Table S19.** Food sources of manganese contribution from cereals and cereal products in the average Polish diet; **Table S20.** Food sources of iodine contribution from cereals and cereal products in the average Polish diet; **Table S21.** Food sources of thiamin contribution from cereals and cereal products in the average Polish diet; **Table S22.** Food sources of riboflavin contribution from cereals and cereal products in the average Polish diet; **Table S23.** Food sources of niacin contribution from cereals and cereal products in the average Polish diet; **Table S24.** Food sources of vitamin B6 contribution from cereals and cereal products in the average Polish diet; **Table S25.** Food sources of folate contribution from cereals and cereal products in the average Polish diet; **Table S26.** Food sources of vitamin B12 contribution from cereals and cereal products in the average Polish diet; **Table S27.** Food sources of vitamin A contribution from cereals and cereal products in the average Polish diet; **Table S28.** Food sources of vitamin D contribution from cereals and cereal products in the average Polish diet; **Table S29.** Food sources of vitamin E contribution from cereals and cereal products in the average Polish diet; **Table S30.** Food sources of vitamin C contribution from cereals and cereal products in the average Polish diet.

## References

[1] McKevith B. (2004). Nutritional aspects of cereals. Br. Nutr. Found. Nutr. Bull..

[2] Kushi L.H., Meyer K.A., Jacobs D.R. (1999). Cereals, legumes, and chronic disease risk reduction: evidence from epidemiologic studies. Am. J. Clin. Nutr..

[3] McIntosh G.H. (2001). Cereal foods, fibres and the prevenation of cancers. Aust. J. Nutr. Diet..

[4] World Health Organization (2003). Diet, Nutrition and the Prevention of Chronic Diseases.

[5] FAO Cereal Supply and Demand Brief. World Food Situation. Cereal production and inventories to decline but overall supplies remain adequate. http://www.fao.org/worldfoodsituation/csdb/en/.

[6] Papanikolaou Y., Fulgoni V.L. (2017). Certain grain foods can be meaningful contributors to nutrient density in the diets of U.S. children and adolescents: Data from the national health and nutrition examination survey, 2009–2012. Nutrients.

[7] O’Neil C.E., Nicklas T.A., Zanovec M., Cho S. (2010). Whole-Grain Consumption Is Associated with Diet Quality and Nutrient Intake in Adults: The National Health and Nutrition Examination Survey, 1999–2004. J. Am. Diet Assoc..

[8] Papanikolaou Y., Fulgoni V. (2018). Grains Contribute Shortfall Nutrients and Nutrient Density to Older US Adults: Data from the National Health and Nutrition Examination Survey, 2011–2014. Nutrients.

[9] Rubio C., Gutiérrez Á.J., Revert C., Reguera J.I., Burgos A., Hardisson A. (2009). Daily dietary intake of iron, copper, zinc and manganese in a Spanish population. Int. J. Food Sci. Nutr..

[10] Yamada M., Asakura K., Sasaki S., Hirota N., Notsu A., Todoriki H., Miura A., Fukui M., Date C. (2014). Estimation of intakes of copper, zinc, and manganese in Japanese adults using 16-day semi-weighed diet records. Asia Pac. J. Clin. Nutr..

[11] Schlemmer U., Frølich W., Prieto R.M., Grases F. (2009). Phytate in foods and significance for humans: Food sources, intake, processing, bioavailability, protective role and analysis. Mol. Nutr. Food Res..

[12] Hunt J.R. (2003). Biaoavailability of iron, zinc, and other trace minerals from vegetarian diets. Am. J. Clin. Nutr..

[13] Sánchez C., López-Jurado M., Aranda P., Llopis J. (2010). Plasma levels of copper, manganese and selenium in an adult population in southern Spain: Influence of age, obesity and lifestyle factors. Sci. Total Environ..

[14] Avila D.S., Puntel R.L., Aschner M. (2013). Manganese in Health and Disease. Interrelations between Essential Metal Ions and Human Diseases.

[15] Chen P., Bornhorst J., Aschner M. (2018). Manganese metabolism in humans. Front. Biosci..

[16] Watts D.L. (1990). The nutritional relationships of manganese. J. Orthomol. Med..

[17] Soetan K.O., Olaiya C.O., Oyewole O.E. (2010). The importance of mineral elements for humans, domestic animals and plants. African J. Food Sci..

[18] Bach Knudsen K.E. (2015). Microbial Degradation of Whole-Grain Complex Carbohydrates and Impact on Short-Chain Fatty. Adv. Nutr..

[19] Ludwig D.S., Hu F.B., Tappy L., Brand-Miller J. (2018). Dietary carbohydrates: Role of quality and quantity in chronic disease. BMJ.

[20] Gellynck X., Kühne B., Van Bockstaele F., Van de Walle D., Dewettinck K. (2009). Consumer perception of bread quality. Appetite.

[21] Aune D., Keum N., Giovannucci E., Fadnes L.T., Boffetta P., Greenwood D.C., Tonstad S., Vatten L.J., Riboli E., Norat T. (2016). Whole grain consumption and risk of cardiovascular disease, cancer, and all cause and cause specific mortality: systematic review and dose-response meta-analysis of prospective studies. BMJ.

[22] Mayor S. (2019). Eating more fibre linked to reduced risk of non-communicable diseases and death, review finds. BMJ.

[23] Reynolds A., Mann J., Cummings J., Winter N., Mete E., Te Morenga L. (2019). Carbohydrate quality and human health: A series of systematic reviews and meta-analyses. Lancet (London, England).

[24] Călinoiu L.F., Vodnar D.C. (2018). Whole Grains and Phenolic Acids: A Review on Bioactivity, Functionality, Health Benefits and Bioavailability. Nutrients.

[25] Huang T., Xu M., Lee A., Cho S., Qi L. (2015). Consumption of whole grains and cereal fiber and total and cause-specific mortality: prospective analysis of 367,442 individuals. BMC Biol..

[26] Kim Y., Je Y. (2014). Dietary fiber intake and total mortality: A meta-analysis of prospective cohort studies. Am. J. Epidemiol..

[27] Zhou X., Xue H., Duan R., Liu Y., Zhang L., Harvey L., Cheng G. (2015). The Cross-Sectional Association of Energy Intake and Dietary Energy Density with Body Composition of Children in Southwest China. Nutrients.

[28] McRae M.P. (2017). Dietary Fiber Is Beneficial for the Prevention of Cardiovascular Disease: An Umbrella Review of Meta-analyses. J. Chiropr. Med..

[29] Seal C.J., Brownlee I.A. (2015). Whole-grain foods and chronic disease: evidence from epidemiological and intervention studies. Proc. Nutr. Soc..

[30] Gani A., Wani S., Masoodi F., Hammed G. (2012). Whole-Grain Cereal Bioactive Compounds and Their Health Benefits: A Review. J. Food Process. Technol..

[31] Tang G., Wang D., Long J., Yang F., Si L. (2015). Meta-Analysis of the Association Between Whole Grain Intake and Coronary Heart Disease Risk. Am. J. Cardiol..

[32] Bechthold A., Boeing H., Schwedhelm C., Hoffmann G., Knüppel S., Iqbal K., De Henauw S., Michels N., Devleesschauwer B., Schlesinger S. (2017). Food groups and risk of coronary heart disease, stroke and heart failure: A systematic review and dose-response meta-analysis of prospective studies. Crit. Rev. Food Sci. Nutr..

[33] McRae M.P. (2017). Health Benefits of Dietary Whole Grains: An Umbrella Review of Meta-analyses. J. Chiropr. Med..

[34] Chen J., Huang Q., Shi W., Yang L., Chen J., Lan Q. (2016). Meta-Analysis of the Association Between Whole and Refined Grain Consumption and Stroke Risk Based on Prospective Cohort Studies. Asia Pacific J. Public Heal..

[35] De Munter J.S., Hu F.B., Spiegelman D., Franz M., van Dam R.M. (2007). Whole Grain, Bran, and Germ Intake and Risk of Type 2 Diabetes: A Prospective Cohort Study and Systematic Review. PLoS Med..

[36] Ley S., Hamdy O., Mohan V., Hu F.B. (2014). Prevention and Management of Type 2 Diabetes: Dietary Components and Nutritional Strategies. Lancet.

[37] Sun Q., Spiegelman D., van Dam R.M., Holmes M.D., Malik V.S., Willett W.C., Hu F.B. (2010). White Rice, Brown Rice, and Risk of Type 2 Diabetes in US Men and Women. Arch. Intern. Med..

[38] Schatzkin A., Park Y., Leitzmann M.F., Hollenbeck A.R., Cross A.J. (2008). Prospective Study of Dietary Fiber, Whole Grain Foods, and Small Intestinal Cancer. Gastroenterology.

[39] Kunzmann A.T., Coleman H.G., Huang W.-Y., Kitahara C.M., Cantwell M.M., Berndt S.I. (2015). Dietary fiber intake and risk of colorectal cancer and incident and recurrent adenoma in the Prostate, Lung, Colorectal, and Ovarian Cancer Screening Trial. Am. J. Clin. Nutr..

[40] Chabowski B.R., Mena J.A., Gonzalez-Padron T.L. (2011). The structure of sustainability research in marketing, 1958–2008: a basis for future research opportunities. J. Acad. Mark. Sci..

[41] Gonzalez C.A., Riboli E. (2010). Diet and cancer prevention: Contributions from the European Prospective Investigation into Cancer and Nutrition (EPIC) study. Eur. J. Cancer.

[42] McRae M.P. (2018). The Benefits of Dietary Fiber Intake on Reducing the Risk of Cancer: An Umbrella Review of Meta-analyses. J. Chiropr. Med..

[43] Aune D., Chan D.S., Lau R., Vieira R., Greenwood D.C., Kampman E., Norat T. (2011). Dietary fibre, whole grains, and risk of colorectal cancer: systematic review and dose-response meta-analysis of prospective studies. BMJ.

[44] Murphy N., Norat T., Ferrari P., Jenab M., Bueno-de-Mesquita B., Skeie G., Dahm C.C., Overvad K., Olsen A., Tjønneland A. (2012). Dietary Fibre Intake and Risks of Cancers of the Colon and Rectum in the European Prospective Investigation into Cancer and Nutrition (EPIC). PLoS One.

[45] Baena R., Salinas P. (2015). Diet and colorectal cancer. Maturitas.

[46] Ben Q., Sun Y., Chai R., Qian A., Xu B., Yuan Y. (2014). Dietary fiber intake reduces risk for colorectal adenoma: a meta-analysis. Gastroenterology.

[47] Song Y., Liu M., Yang F.G., Cui L.H., Lu X.Y., Chen C. (2015). Dietary fibre and the risk of colorectal cancer: a case- control study. Asian Pac. J. Cancer Prev..

[48] Wiseman M., Thompson R. (2018). Diet, Nutrition, Physical Activity and Cancer: A Global Perspective.

[49] Popkin B. (2002). An overview on the nutrition transition and its health implications: the Bellagio meeting. Public Health Nutr..

[50] Smil V. (2000). Feeding the World: A Challenge for the Twenty-First Century.

[51] Kearney J. (2010). Food consumption trends and drivers. Philos. Trans. R. Soc. B Biol. Sci..

[52] Drewnowski A., Popkin B.M. (1997). The nutrition transition: new trends in the global diet. Nutr. Rev..

[53] Willett W., Rockström J., Loken B., Springmann M., Lang T., Vermeulen S., Garnett T., Tilman D., DeClerck F., Wood A. (2019). Food in the Anthropocene: the healthy diets from sustainable food systems. Summ. Rep. EAT-Lancet Comm..

[54] Central Statistical Office (2017). Household Budget Survey in 2016.

[55] Barlik M., Siwiak K., Central Statistical Office (2011). Methodology of Household Survey (in Polish).

[56] Laskowski W., Górska-Warsewicz H., Kulykovets O. (2018). Meat, Meat Products and Seafood as Sources of Energy and Nutrients in the Average Polish Diet. Nutrients.

[57] Górska-Warsewicz H., Laskowski W., Kulykovets O., Kudlińska-Chylak A., Czeczotko M., Rejman K. (2018). Food Products as Sources of Protein and Amino Acids—The Case of Poland. Nutrients.

[58] Laskowski W., Górska-Warsewicz H. (2014). Nutrient Density of the Average Polish Diet. Economic Analysis.

[59] Kunachowicz H., Przygoda B., Nadolna I., Iwanow K. (2017). Tabele Składu i Wartości Odżywczej Żywności.

[60] Fox J., Leanage A. (2016). R and the Journal of Statistical Software. J. Stat. Softw..

[61] R Development Core Team R Language Definition. https://cran.r-project.org/doc/manuals/r-release/R-lang.pdf.

[62] Lang M. (2017). Efficient *R* Programming. J. Stat. Softw..

[63] Rejman K., Kowrygo B., Laskowski W. (2015). Evaluation of the Structure of Food Consumption in Poland in the Context of Demands of Sustainable Consumption (in Polish). J. Agribus. Rural Dev..

[64] Jarosz M. (2017). Reference Energy and Nutrient Intake for the Polish Population (in Polish).

[65] What We Eat in America. https://www.ars.usda.gov/ARSUserFiles/80400530/pdf/1314/Food_categories_2013-2014.pdf.

[66] O’Neil C.E., Keast D.R., Fulgoni V.L., Nicklas T.A. (2012). Food sources of energy and nutrients among adults in the US: NHANES 2003-2006. Nutrients.

[67] Scientific Report of the 2015 Dietary Guidelines Advisory Committee Appendix E-2.7: Major Categories and Subcategories used in DGAC Analyses of WWEIA Food Categories Part E. Section 2: Supplementary Documentation to the 2015 DGAC Report Scie. https://health.gov/dietaryguidelines/DGAC-Major-categories-and-subcategories-from-WWEIA-FoodCategories.pdf.

[68] Rhodes D.G., Adler M.E., Clemens J.C., Moshfegh A.J. (2017). What we eat in America food categories and changes between survey cycles. J. Food Compos. Anal..

[69] Górska-Warsewicz H., Świątkowska M., Jeżewska-Zychowicz M. (2015). Consumption of Bread and other Cereals Products in the Polish Households. Innovative Cereal Products for the Consumer Perspective (in Polish).

[70] Alexandratos N., Bruinsma J. (2012). Worlds Agriculture Towards 2030/2050: the 2012 Revision. Working Paper No. 12.03.

[71] Nagyová Ľ., Rovný P., Stávková J., Uličná M., Maďarová Ľ. (2009). Consumer perception of bread quality. Acta Univ. Agric. Silvic. Mendelianae Brun..

[72] Central Statistical Office (2013). Household Budget Survey in 2013.

[73] Steer T., Thane C., Stephen A., Jebb S. (2008). Bread in the diet: consumption and contribution to nutrient intakes of British adults. Proc. Nutr. Soc..

[74] Roberts C., Steer T., Maplethorpe N., Cox L., Meadows S., Nicholson S., Page P., Swan G. (2018). National Diet and Nutrition Survey: Results from Years 7 and 8 (combined) of the Rolling Programme (2014/2015 to 2015/2016) Public Health England. https://assets.publishing.service.gov.uk/government/uploads/system/uploads/attachment_data/file/699241/NDNS_results_years_7_and_8.pdf.

[75] Mielgo-Ayuso J., Aparicio-Ugarriza R., Olza J., Aranceta-Bartrina J., Gil Á., Ortega R., Serra-Majem L., Varela-Moreiras G., González-Gross M. (2018). Dietary Intake and Food Sources of Niacin, Riboflavin, Thiamin and Vitamin B6 in a Representative Sample of the Spanish Population. The Anthropometry, Intake, and Energy Balance in Spain (ANIBES) Study †. Nutrients.

[76] Piwowar A. (2017). Consumption of Basic Products of Vegetable and Animal Origin in Poland in 2000-2012 (in Polish). Handel Wewnętrzny.

[77] Sandvik P., Kihlberg I., Lindroos A.K., Marklinder I., Nydahl M. (2014). Bread consumption patterns in a Swedish national dietary survey focusing particularly on whole-grain and rye bread. Food Nutr. Res..

[78] Goryńska-Goldman E. (2010). Tendencies of bread consumption in Poland (in Polish). Sci. Pol. Oeconomica.

[79] Borowska A., Rejman K. (2009). The Use of Nutrition and Health Information on the Bakery Market to Increase the Demand for its Products (in Polish). Med. Sport. Pract..

[80] Borowska A., Rejman K. (2011). Bread Consumption and Consumer Preferences Considering Product Innovation of Bakery Sector (in Polish). Stud. i Mater. Zarządzania Wiedzą.

[81] Central Statistical Office (2018). Statistical Yearbook of the Republic of Poland.

[82] Heiniö R.L., Noort M.W.J., Katina K., Alam S.A., Sozer N., de Kock H.L., Hersleth M., Poutanen K. (2016). Sensory characteristics of wholegrain and bran-rich cereal foods—A review. Trends Food Sci. Technol..

[83] Heenan S.P., Dufour J.-P., Hamid N., Harvey W., Delahunty C.M. (2008). The sensory quality of fresh bread: Descriptive attributes and consumer perceptions. Food Res. Int..

[84] Heenan S.P., Hamid N., Dufour J.-P., Harvey W., Delahunty C.M. (2009). Consumer freshness perceptions of breads, biscuits and cakes. Food Qual. Prefer..

[85] Heenan S.P., Dufour J.-P., Hamid N., Harvey W., Delahunty C.M. (2009). Characterisation of fresh bread flavour: Relationships between sensory characteristics and volatile composition. Food Chem..

[86] Bernstein A.J., Rose D.J. (2015). Preference Mapping of Commercial Whole Wheat Breads. Cereal Chem. J..

[87] Ha C.-H., Lee S.M., Lee E.-K., Kim K.-O. (2017). Effect of flour information (origin and organic) and consumer attitude to health and natural product on bread acceptability of Korean consumers. J. Sens. Stud..

[88] Pasiakos S.M., Agarwal S., Lieberman H.R., Fulgoni V.L. (2015). Sources and amounts of animal, dairy, and plant protein intake of US adults in 2007–2010. Nutrients.

[89] Hamułka J., Wawrzyniak A., Sosińska S. (2008). Evaluation of Dietary Fibre, Soluble and Insoluble Fibre Food Intake in Polish Households in 1996-2005 (in Polish). Rocz. Państwowego Zakładu Hig..

[90] Górecka D., Janus P., Borysiak-Marzec P., Dziedzic K. (2011). Analysis of Consumption of Dietary Fibre and its Fractions in Poland in Last Decade based on the Statistical Yearbook Data (in Polish). Probl Hig Epidemio.

[91] (2001). Dietary Reference Intakes for Vitamin A, Vitamin K, Arsenic, Boron, Chromium, Copper, Iodine, Iron, Manganese, Molybdenum, Nickel, Silicon, Vanadium, and Zinc.

[92] Brzozowska A., Gawęcki J. (2010). Microminerals. Human Nutrition. Th Base of Nutrition (in Polish).

[93] Lonnerdal B. (2002). Phytic acid-trace element (Zn, Cu, Mn) interactions. Int. J. Food Sci. Technol..

[94] Hurrell R.F. (2003). Influence of Vegetable Protein Sources on Trace Element and Mineral Bioavailability. J. Nutr..

[95] Gawęcki J., Woźniewicz M., Gawęcki J. (2010). Bread and cereal products. Human Nutrition. Th Base of Nutrition (in Polish).

[96] Manganese. Linus Pauling Institute. https://lpi.oregonstate.edu/mic/minerals/manganese.

[97] Hope S.J., Daniel K., Gleason K.L., Comber S., Nelson M., Powell J.J. (2005). Influence of tea drinking on manganese intake, manganese status and leucocyte expression of MnSOD and cytosolic aminopeptidase P. Eur. J. Clin. Nutr..

[98] Podwika W., Kleszcz K., Krośniak M., Zagrodzki P. (2017). Copper, Manganese, Zinc, and Cadmium in Tea Leaves of Different Types and Origin. Biol. Trace Elem. Res..

[99] Polechońska L., Dambiec M., Klink A., Rudecki A. (2015). Concentrations and solubility of selected trace metals in leaf and bagged black teas commercialized in Poland. J. Food Drug Anal..

[100] Samaniego-Vaesken L., Partearroyo T., Olza J., Aranceta-Bartrina J., Gil Á., González-Gross M., Ortega R.M., Serra-Majem L., Varela-Moreiras G. (2017). Iron Intake and Dietary Sources in the Spanish Population: Findings from the ANIBES Study. Nutrients.

[101] Rehm C.D., Drewnowski A. (2017). Replacing American breakfast foods with ready-to-eat (RTE) cereals increases consumption of key food groups and nutrients among US children and adults: Results of an NHANES modeling study. Nutrients.

[102] Deshmukh-Taskar P.R., Nicklas T.A., O’Neil C.E., Keast D.R., Radcliffe J.D., Cho S. (2010). The Relationship of Breakfast Skipping and Type of Breakfast Consumption with Nutrient Intake and Weight Status in Children and Adolescents: The National Health and Nutrition Examination Survey 1999-2006. J. Am. Diet. Assoc..

[103] Drewnowski A., Rehm C., Vieux F. (2018). Breakfast in the United States: Food and Nutrient Intakes in Relation to Diet Quality in National Health and Examination Survey 2011–2014. A Study from the International Breakfast Research Initiative. Nutrients.

[104] Walker P., Rhubart-Berg P., Mckenzie S., Kelling K., Lawrence R.S. (2005). Public health implications of meat production and consumption. Public Health Nutr..

[105] Richi E.B., Baumer B., Conrad B., Darioli R., Schmid A., Keller U. (2015). Health Risks Associated with Meat Consumption: A Review of Epidemiological Studies. Int. J. Vitam. Nutr. Res.

[106] Sharma S., Sheehy T., Kolonel L.N. (2013). Contribution of meat to vitamin B _12_, iron and zinc intakes in five ethnic groups in the USA: implications for developing food-based dietary guidelines. J. Hum. Nutr. Diet..

[107] Wyness L. (2016). The role of red meat in the diet: Nutrition and health benefits. Proc. Nutr. Soc..

[108] Pilis W., Stec K., Zych M., Pilis A. (2014). Health benefits and risk associated with adopting a vegetarian diet. Rocz. Państwowego Zakładu Hig..

[109] Marsk K.A., Munn E.A., Baines S.K. (2013). Protein and vegetarian diets. Med. J. Aust..

[110] (2009). Position of the American Dietetic Association: Vegetarian Diets. J. Am. Diet. Assoc..

[111] Finnamore H.E., Whelan K., Hickson M., Shovlin C.L. (2014). Top dietary iron sources in the UK. Br. J. Gen. Pract..

[112] Andrade G.C., da Costa Louzada M.L., Azeredo C.M., Ricardo C.Z., Martins A.P.B., Levy R.B. (2018). Out-of-home food consumers in Brazil: What do they eat?. Nutrients.

[113] Troesch B., Biesalski H.K., Bos R., Buskens E., Calder P.C., Saris W.H.M., Spieldenner J., Verkade H.J., Weber P., Eggersdorfer M. (2015). Increased intake of foods with high nutrient density can help to break the intergenerational cycle of malnutrition and obesity. Nutrients.

[114] Denney L., Afeiche M.C., Eldridge A.L., Villalpando-Carrión S. (2017). Food sources of energy and nutrients in infants, toddlers, and young children from the Mexican National Health and Nutrition Survey 2012. Nutrients.

[115] Sicińska E., Wyka J. (2011). Folate Intake in Poland on the Basis of Literature (in Polish). Rocz. Państwowego Zakładu Hig..

[116] Drewnowski A. (2005). Concept of a nutritious food: toward a nutrient density score1–3. Am. J. Clin. Nutr..

[117] Jarosz M. Pyramid of Healthy Eating and Physical Activity for Adults (in Polish). https://ncez.pl/abc-zywienia-/zasady-zdrowego-zywienia/piramida-zdrowego-zywienia-i-aktywnosci-fizycznej-dla-osob-doroslych.

[118] A Healthy and Sustainable Food Future: Policy recommendations to embed sustainability in the Eatwell Guide and wider UK food policy. https://www.eating-better.org/uploads/Documents/AHealthySustainableFoodFuture(1).pdf.

