# Supplementary material for: How Important are Cereals and Cereal Products in the Average Polish Diet?
Source: Nutrients. 2019 Mar 21;11(3):679. doi: 10.3390/nu11030679 (PMC6470554; doi:10.3390/nu11030679)
Supplement: Supplementary file 1 [file nutrients-11-00679-s001.pdf]

How Important are Cereals and Cereal Products in the Average Polish Diet?

Supplemental Tables

Table S1. Food grouping for the purpose of energy and nutrient source analysis
Table S2. Food sources of energy contribution from cereals and cereal products in the average Polish diet
Table S3. Food sources of carbohydrates contribution from cereals and cereal products in the average Polish diet
Table S4. Food sources of protein contribution from cereals and cereal products in the average Polish diet
Table S5. Food sources of total fat contribution from cereals and cereal products in the average Polish diet
Table S6. Food sources of SFA contribution from cereals and cereal products in the average Polish diet
Table S7. Food sources of MUFA contribution from cereals and cereal products in the average Polish diet
Table S8. Food sources of PUFA contribution from cereals and cereal products in the average Polish diet
Table S9. Food sources of cholesterol contribution from cereals and cereal products in the average Polish diet
Table S10. Food sources of dietary fibre contribution from cereals and cereal products in the average Polish diet
Table S11. Food sources of calcium contribution from cereals and cereal products in the average Polish diet
Table S12. Food sources of phosphorus contribution from cereals and cereal products in the average Polish diet
Table S13. Food sources of sodium contribution from cereals and cereal products in the average Polish diet
Table S14. Food sources of potassium contribution from cereals and cereal products in the average Polish diet
Table S15. Food sources of magnesium contribution from cereals and cereal products in the average Polish diet
Table S16. Food sources of iron contribution from cereals and cereal products in the average Polish diet
Table S17. Food sources of zinc contribution from cereals and cereal products in the average Polish diet
Table S18. Food sources of copper contribution from cereals and cereal products in the average Polish diet
Table S19. Food sources of manganese contribution from cereals and cereal products in the average Polish diet
Table S20. Food sources of iodine contribution from cereals and cereal products in the average Polish diet
Table S21. Food sources of thiamin contribution from cereals and cereal products in the average Polish diet
Table S22. Food sources of riboflavin contribution from cereals and cereal products in the average Polish diet
Table S23. Food sources of niacin contribution from cereals and cereal products in the average Polish diet
Table S24. Food sources of vitamin B6 contribution from cereals and cereal products in the average Polish diet
Table S25. Food sources of folate contribution from cereals and cereal products in the average Polish diet

Table S26. Food sources of vitamin B12 contribution from cereals and cereal products in the average Polish diet
Table S27. Food sources of vitamin A contribution from cereals and cereal products in the average Polish diet
Table S28. Food sources of vitamin D contribution from cereals and cereal products in the average Polish diet
Table S29. Food sources of vitamin E contribution from cereals and cereal products in the average Polish diet
Table S30. Food sources of vitamin C contribution from cereals and cereal products in the average Polish diet

Table S1. Food grouping for the purpose of energy and nutrient source analysis

Food Categories (13)	Main Groups (42)	Sub-groups (91)
GRAIN PRODUCTS	bread, rolls, bread products	bread and rolls<br>quick breads and bread products
	rice, cooked grains	rice<br>groats and cereal grains
	flour, bran, cooking ingredients	wheat flour<br>other flours
	pizza, pasta, and other flour dishes	pasta, macaroni, noodles<br>pizza and other flour dishes
	ready-to-eat cereal	breakfast cereals
MEAT AND MEAT PRODUCTS	red meat	beef<br>pork<br>sheep, goat<br>veal
	meat products	processed red meat products<br>processed poultry meat products<br>other meat products
	other meat	liver and organ meat<br>minced meat<br>other meat
	poultry	chicken<br>poultry excluding chicken
MILK AND DAIRY PRODUCTS	milk	whole milk<br>reduced fat milk<br>condensed and powdered milk
	cheese	cheeses
	cottage cheese	cottage cheese
	yoghurts and milk drinks	yogurt,<br>milk shakes and other dairy drinks
SEAFOOD	fish	fresh, chilled or frozen fish
	shellfish	fresh, chilled or frozen shellfish
	processed seafood	dried, smoked and salted seafood<br>other fish and shellfish products
EGGS	eggs	eggs
FATS AND OILS	butter	butter
	oils, olive	olive oil<br>other oils
	other fats	margarine and other plant fats<br>other animal fats
	sour cream	cream
FRUITS	fruits	apples<br>bananas<br>berries<br>citrus fruits<br>frozen fruits<br>fruits products<br>other fruits<br>peaches and nectarines

	dried fruits and nuts	dried fruits and nuts
VEGETABLES	potatoes	potatoes<br>potatoes products
	vegetables (excluding potatoes)	beetroot<br>cabbage<br>carrots<br>cauliflower<br>cucumber<br>lettuce<br>onions<br>tomatoes<br>frozen vegetables and mushrooms<br>sour cabbage<br>other vegetables and mushrooms<br>vegetable and mushrooms products
SNACKS AND SWEETS	chocolate	chocolate<br>powdered cacao<br>powdered chocolate
	desserts	ice-cream
	snacks	chips
	sweet bakery products	cakes and pies
SUGARS AND SALT	honey	honey
	jams, syrups, marmalade	jams<br>syrups<br>marmalade
	sugar	sugar
	sugar substitutes	sugar substitutes
	salt	salt
BEVERAGES, NONALCOHOLIC	juices	fruit juices<br>vegetables and mixed juices
	other beverages	other nonalcoholic beverages
	water	water
COFFEE, TEA	coffee	coffee
	tea	tea
ALCOHOLIC BEVERAGES	wine	grape wine<br>high alcohol wine<br>other wine<br>wine-based beverages
	beer	beer lager<br>low-alcohol and non-alcohol beer<br>other beer<br>beer-based beverages
	other alcoholic beverages	liquor and cocktails<br>other alcoholic beverages

Table S2. Food sources of energy contribution (%) from cereals and cereal products in the average Polish diet

Main Group<br>Sub- group	Rank	% of Energy Contribution	Cumulative % of Energy Contribution
**Main Group**			
bread, rolls, bread products	1	21.92	21.92
flour, bran, cooking ingredients	2	3.37	25.29
pizza, pasta and other flour dishes	3	2.60	27.89
rice, cooked grains	4	1.56	29.44
ready-to-eat cereal	5	0.96	30.40
**Sub- group**			
bread and rolls	1	16.44	16.44
quick breads and other bread products	2	5.48	21.92
wheat flour	3	3.30	25.22
pasta, macaroni, noodles	4	2.09	27.30
breakfast cereals	5	0.96	28.26
rice	6	0.89	29.14
groats and cereal grains	7	0.67	29.81
pizza and other flour dishes	8	0.51	30.33
other flours	9	0.07	30.40

Table S3. Food sources of carbohydrates contribution from cereals and cereal products in the average Polish diet

Main Group<br>Sub-group	Rank	% of Carbohydrates Contribution	Cumulative % of Carbohydrates Contribution
**Main Group**			
bread, rolls, bread products	1	36.33	36.33
flour, bran, cooking ingredients	2	5.92	42.25
pizza, pasta and other flour dishes	3	4.21	46.46
rice, cooked grains	4	2.89	49.35
ready-to-eat cereal	5	1.62	50.97
**Sub-group**			
bread and rolls	1	28.76	28.76
quick breads and other bread products	2	7.57	36.33
wheat flour	3	5.78	42.12
pasta, macaroni, noodles	4	3.69	45.81
rice	5	1.70	47.51
breakfast cereals	6	1.62	49.13
groats and cereal grains	7	1.19	50.32
pizza and other flour dishes	8	0.52	50.83
other flours	9	0.13	50.97

Table S4. Food sources of protein contribution from cereals and cereal products in the average Polish diet

Main Group<br>Sub-group	Rank	% of Protein Contribution	Cumulative % of Protein Contribution
**Main Group**			
bread, rolls, bread products	1	16.49	16.49
flour, bran, cooking ingredients	2	3.10	19.59
pizza, pasta and other flour dishes	3	2.47	22.07
rice, cooked grains	4	1.09	23.16
ready-to-eat cereal	5	0.77	23.93
**Sub-group**			
bread and rolls	1	13.22	13.22
quick breads and other bread products	2	3.27	16.49
wheat flour	3	3.05	19.54
pasta, macaroni, noodles	4	1.84	21.38
breakfast cereals	5	0.77	22.16
pizza and other flour dishes	6	0.63	22.79
groats and cereal grains	7	0.58	23.37
rice	8	0.51	23.88
other flours	9	0.05	23.93

Table S5. Food sources of total fat contribution from cereals and cereal products in the average Polish diet

Main Group<br>Sub-group	Rank	% of Total Fat Contribution	Cumulative % of Total Fat Contribution
**Main Group**			
bread, rolls, bread products	1	8.12	8.12
pizza, pasta and other flour dishes	2	0.84	8.97
flour, bran, cooking ingredients	3	0.37	9.34
ready-to-eat cereal	4	0.34	9.68
rice, cooked grains	5	0.19	9.87
**Sub-group**			
quick breads and other bread products	1	4.15	4.15
bread and rolls	2	3.97	8.12
pizza and other flour dishes	3	0.51	8.63
wheat flour	4	0.36	8.99
breakfast cereals	5	0.34	9.33
pasta, macaroni, noodles	6	0.34	9.66
groats and cereal grains	7	0.12	9.78
rice	8	0.07	9.86
other flours	9	0.01	9.87

Table S6. Food sources of SFA contribution from cereals and cereal products in the average Polish diet

Main Group<br>Sub-group	Rank	% of SFA Contribution	Cumulative % of SFA Contribution
**Main Group**			
bread, rolls, bread products	1	6.91	6.91
pizza, pasta and other flour dishes	2	0.76	7.67
flour, bran, cooking ingredients	3	0.24	7.91
ready-to-eat cereal	4	0.17	8.08
rice, cooked grains	5	0.11	8.19
**Sub-group**			
quick breads and other bread products	1	4.56	4.56
bread and rolls	2	2.35	6.91
pizza and other flour dishes	3	0.53	7.44
wheat flour	4	0.23	7.68
pasta, macaroni, noodles	5	0.23	7.91
breakfast cereals	6	0.17	8.07
groats and cereal grains	7	0.06	8.13
rice	8	0.05	8.18
other flours	9	0.01	8.19

Table S7. Food sources of MUFA contribution from cereals and cereal products in the average Polish diet

Main Group<br>Sub-group	Rank	% of MUFA Contribution	Cumulative % of MUFA Contribution
**Main Group**			
bread, rolls, bread products	1	7.71	7.71
pizza, pasta and other flour dishes	2	0.79	8.49
ready-to-eat cereal	3	0.34	8.83
flour, bran, cooking ingredients	4	0.13	8.97
rice, cooked grains	5	0.11	9.08
**Sub-group**			
quick breads and other bread products	1	4.31	4.31
bread and rolls	2	3.39	7.71
pizza and other flour dishes	3	0.53	8.23
breakfast cereals	4	0.34	8.57
pasta, macaroni, noodles	5	0.26	8.83
wheat flour	6	0.13	8.96
groats and cereal grains	7	0.06	9.02
rice	8	0.05	9.07

Table S8. Food sources of PUFA contribution from cereals and cereal products in the average Polish diet

Main Group<br>Sub-group	Rank	% of PUFA Contribution	Cumulative % of PUFA Contribution
**Main Group**			
bread, rolls, bread products	1	13.63	13.63
flour, bran, cooking ingredients	2	1.35	14.98
pizza, pasta and other flour dishes	3	1.22	16.20
ready-to-eat cereal	4	0.74	16.93
rice, cooked grains	5	0.46	17.39
**Sub-group**			
bread and rolls	1	10.07	10.07
quick breads and other bread products	2	3.56	13.63
wheat flour	3	1.32	14.95
breakfast cereals	4	0.74	15.69
pasta, macaroni, noodles	5	0.73	16.42
pizza and other flour dishes	6	0.48	16.90
groats and cereal grains	7	0.30	17.20
rice	8	0.16	17.36
other flours	9	0.03	17.39

Table S9. Food sources of cholesterol contribution from cereals and cereal products in the average Polish diet

Main Group<br>Sub-group	Rank	% of Cholesterol Contribution	Cumulative % of Cholesterol Contribution
**Main Group**			
bread, rolls, bread products	1	4.19	4.19
pizza, pasta and other flour dishes	2	2.19	6.38
**Sub-group**			
quick breads and other bread products	1	3.54	3.54
pasta, macaroni, noodles	2	1.57	5.11
bread and rolls	3	0.66	5.76
pizza and other flour dishes	4	0.62	6.38

Table S10. Food sources of dietary fibre contribution from cereals and cereal products in the average Polish diet

Main Group<br>Sub-group	Rank	% of Dietary Fibre Contribution	Cumulative % of Dietary Fibre Contribution
**Food Group**			
bread, rolls, bread products	1	35.43	35.43
flour, bran, cooking ingredients	2	4.35	39.77
ready-to-eat cereal	3	3.19	42.97
rice, cooked grains	4	2.91	45.88
pizza, pasta and other flour dishes	5	2.57	48.45
**Sub-group**			
bread and rolls	1	31.29	31.29
wheat flour	2	4.17	35.46
quick breads and other bread products	3	4.14	39.60
breakfast cereals	4	3.19	42.79
pasta, macaroni, noodle	5	2.19	44.98
rice	6	1.48	46.46
groats and cereal grains	7	1.44	47.90
pizza and other flour dishes	8	0.38	48.27
other flours	9	0.17	48.45

Table S11. Food sources of calcium contribution from cereals and cereal products in the average Polish diet

Main Group<br>Sub-group	Rank	% of Calcium Contribution	Cumulative % of Calcium Contribution
**Main Group**			
bread, rolls, bread products	1	8.76	8.76
pizza, pasta and other flour dishes	2	1.43	10.19
flour, bran, cooking ingredients	3	0.82	11.01
ready-to-eat cereal	4	0.62	11.63
rice, cooked grains	5	0.32	11.95
**Sub-group**			
bread and rolls	1	6.48	6.48
quick breads and other bread products	2	2.29	8.76
pizza and other flour dishes	3	0.91	9.67
wheat flour	4	0.79	10.46
breakfast cereals	5	0.62	11.08
pasta, macaroni, noodles	6	0.52	11.60
groats and cereal grains	7	0.17	11.77
rice	8	0.15	11.92
other flours	9	0.03	11.95

Table S12. Food sources of phosphorus contribution from cereals and cereal products in the average Polish diet

Main Group<br>Sub-group	Rank	% of Phosphorus Contribution	Cumulative % of Phosphorus Contribution
**Main Group**			
bread, rolls, bread products	1	17.56	17.56
flour, bran, cooking ingredients	2	2.05	19.61
pizza, pasta and other flour dishes	3	2.02	21.63
rice, cooked grains	4	1.84	23.46
ready-to-eat cereal	5	1.53	24.99
**Sub-group**			
bread and rolls	1	14.77	14.77
quick breads and other bread products	2	2.79	17.56
wheat flour	3	1.97	19.53
breakfast cereals	4	1.53	21.06
pasta, macaroni, noodles	5	1.45	22.51
groats and cereal grains	6	1.04	23.55
rice	7	0.80	24.34
pizza and other flour dishes	8	0.57	24.91
other flours	9	0.08	24.99

Table S13. Food sources of sodium contribution from cereals and cereal products in the average Polish diet

Main Group<br>Sub-group	Rank	% of Sodium Contribution	Cumulative % of Sodium Contribution
**Main Group**			
bread, rolls, bread products	1	17.50	17.50
ready-to-eat cereal	2	0.49	17.99
pizza, pasta and other flour dishes	3	0.36	18.36
rice, cooked grains	4	0.02	18.38
flour, bran, cooking ingredients	5	0.01	18.39
**Sub-group**			
bread and rolls	1	15.24	15.24
quick breads and other bread products	2	2.26	17.50
breakfast cereals	3	0.49	17.99
pizza and other flour dishes	4	0.31	18.30
pasta, macaroni, noodles	5	0.05	18.36
wheat flour	6	0.01	18.37
rice	7	0.01	18.38
groats and cereal grains	8	0.01	18.39

Table S14. Food sources of potassium contribution from cereals and cereal products in the average Polish diet

Main Group<br>Sub-group	Rank	% of Potassium Contribution	Cumulative % of Potassium Contribution
**Main Group**			
bread, rolls, bread products	1	12.19	12.19
pizza, pasta and other flour dishes	2	1.37	13.56
flour, bran, cooking ingredients	3	1.35	14.91
rice, cooked grains	4	0.87	15.78
ready-to-eat cereal	5	0.85	16.64
**Sub-group**			
bread and rolls	1	10.11	10.11
quick breads and other bread products	2	2.08	12.19
wheat flour	3	1.31	13.50
pasta, macaroni, noodles	4	1.01	14.51
breakfast cereals	5	0.85	15.36
groats and cereal grains	6	0.53	15.89
pizza and other flour dishes	7	0.36	16.25
rice	8	0.35	16.59
other flours	9	0.04	16.64

Table S15. Food sources of magnesium contribution from cereals and cereal products in the average Polish diet

Main Group<br>Sub-group	Rank	% of Magnesium Contribution	Cumulative % of Magnesium Contribution
**Main Group**			
bread, rolls, bread products	1	20.97	20.97
rice, cooked grains	2	2.68	23.65
ready-to-eat cereal	3	2.48	26.13
flour, bran, cooking ingredients	4	2.07	28.20
pizza, pasta and other flour dishes	5	2.03	30.23
**Sub-group**			
bread and rolls	1	18.11	18.11
quick breads and other bread products	2	2.86	20.97
breakfast cereals	3	2.48	23.45
wheat flour	4	1.95	25.39
groats and cereal grains	5	1.77	27.16
pasta, macaroni, noodles	6	1.71	28.87
rice	7	0.91	29.79
pizza and other flour dishes	8	0.32	30.10
other flours	9	0.12	30.23

Table S16. Food sources of iron contribution from cereals and cereal products in the average Polish diet

Main Group<br>Sub-group	Rank	% of Iron Contribution	Cumulative % of Iron Contribution
**Main Group**			
bread, rolls, bread products	1	24.93	24.93
flour, bran, cooking ingredients	2	3.26	28.18
ready-to-eat cereal	3	2.98	31.17
pizza, pasta and other flour dishes	4	2.24	33.41
rice, cooked grains	5	1.73	35.14
**Sub-group**			
bread and rolls	1	20.35	20.35
quick breads and other bread products	2	4.58	24.93
wheat flour	3	3.14	28.07
breakfast cereals	4	2.98	31.05
pasta, macaroni, noodles	5	1.67	32.73
groats and cereal grains	6	1.22	33.95
pizza and other flour dishes	7	0.56	34.51
rice	8	0.52	35.02
other flours	9	0.11	35.14

Table S17. Food sources of zinc contribution from cereals and cereal products in the average Polish diet

Main Group<br>Sub-group	Rank	% of Zinc Contribution	Cumulative % of Zinc Contribution
**Main Group**			
bread, rolls, bread products	1	21.12	21.12
flour, bran, cooking ingredients	2	2.32	23.44
rice, cooked grains	3	2.06	25.50
pizza, pasta and other flour dishes	4	1.75	27.24
ready-to-eat cereal	5	1.36	28.60
**Sub-group**			
bread and rolls	1	18.59	18.59
quick breads and other bread products	2	2.52	21.12
wheat flour	3	2.23	23.35
breakfast cereals	4	1.36	24.70
pasta, macaroni, noodles	5	1.29	25.99
groats and cereal grains	6	1.11	27.11
rice	7	0.95	28.05
pizza and other flour dishes	8	0.45	28.51
other flours	9	0.09	28.60

Table S18. Food sources of copper contribution from cereals and cereal products in the average Polish diet

Main Group<br>Sub-group	Rank	% of Copper Contribution	Cumulative % of Copper Contribution
**Main Group**			
bread, rolls, bread products	1	22.14	22.14
flour, bran, cooking ingredients	2	2.59	24.73
rice, cooked grains	3	2.44	27.18
pizza, pasta and other flour dishes	4	2.22	29.40
ready-to-eat cereal	5	1.87	31.26
**Sub-group**			
bread and rolls	1	18.91	18.91
quick breads and other bread products	2	3.24	22.14
wheat flour	3	2.51	24.65
breakfast cereals	4	1.87	26.52
pasta, macaroni, noodles	5	1.84	28.36
groats and cereal grains	6	1.43	29.78
rice	7	1.02	30.80
pizza and other flour dishes	8	0.38	31.18
other flours	9	0.08	31.26

Table S19. Food sources of manganese contribution from cereals and cereal products in the average Polish diet

Main Group<br>Sub-group	Rank	% of Manganese Contribution	Cumulative % of Manganese Contribution
**Main Group**			
bread, rolls, bread products	1	48.55	48.55
rice, cooked grains	2	4.54	53.09
ready-to-eat cereal	3	4.41	57.50
flour, bran, cooking ingredients	4	3.98	61.47
pizza, pasta and other flour dishes	5	2.58	64.05
**Sub-group**			
bread and rolls	1	45.11	45.11
breakfast cereals	2	4.41	49.52
wheat flour	3	3.76	53.28
quick breads and other bread products	4	3.44	56.72
rice	5	2.43	59.15
pasta, macaroni, noodles	6	2.30	61.45
groats and cereal grains	7	2.11	63.56
pizza and other flour dishes	8	0.28	63.84
other flours	9	0.22	64.05

Table S20. Food sources of iodine contribution from cereals and cereal products in the average Polish diet

**Main Group<br>Sub-group**	**Rank**	**% of Iodine Contribution**	**Cumulative % of Iodine Contribution**
**Main Group**			
bread, rolls, bread products	1	5.59	5.59
flour, bran, cooking ingredients	2	0.32	5.91
ready-to-eat cereal	3	0.28	6.19
rice, cooked grains	4	0.22	6.41
pizza, pasta and other flour dishes	5	0.20	6.61
**Sub-group**			
bread and rolls	1	5.37	5.37
wheat flour	2	0.30	5.67
breakfast cereals	3	0.28	5.95
quick breads and other bread products	4	0.22	6.18
pasta, macaroni, noodles	5	0.17	6.35
groats and cereal grains	6	0.11	6.46
rice	7	0.11	6.57
pizza and other flour dishes	8	0.03	6.60
bread and rolls	1	5.37	5.37

Table S21. Food sources of thiamin contribution from cereals and cereal products in the average Polish diet

Main Group<br>Sub-group	Rank	% of Thiamin Contribution	Cumulative % of Thiamin Contribution
**Main Group**			
bread, rolls, bread products	1	17.42	17.42
flour, bran, cooking ingredients	2	4.53	21.95
rice, cooked grains	3	2.08	24.03
ready-to-eat cereal	4	2.05	26.07
pizza, pasta and other flour dishes	5	1.93	28.01
**Sub-group**			
bread and rolls	1	14.93	14.93
wheat flour	2	4.45	19.37
quick breads and other bread products	3	2.50	21.87
breakfast cereals	4	2.05	23.92
pasta, macaroni, noodles	5	1.59	25.50
groats and cereal grains	6	1.33	26.83
rice	7	0.75	27.58
pizza and other flour dishes	8	0.35	27.93
other flours	9	0.08	28.01

Table S22. Food sources of riboflavin contribution from cereals and cereal products in the average Polish diet

Main Group<br>Sub-group	Rank	% of Riboflavin Contribution	Cumulative % of Riboflavin Contribution
**Main Group**			
bread, rolls, bread products	1	11.19	11.19
flour, bran, cooking ingredients	2	2.76	13.95
pizza, pasta and other flour dishes	3	1.48	15.42
ready-to-eat cereal	4	1.13	16.56
rice, cooked grains	5	0.53	17.08
**Sub-group**			
bread and rolls	1	8.69	8.69
wheat flour	2	2.70	11.40
quick breads and other bread products	3	2.50	13.89
breakfast cereals	4	1.13	15.03
pasta, macaroni, noodles	5	0.94	15.97
pizza and other flour dishes	6	0.53	16.50
groats and cereal grains	7	0.41	16.91
rice	8	0.12	17.03
other flours	9	0.06	17.08

Table S23. Food sources of niacin contribution from cereals and cereal products in the average Polish diet

Main Group<br>Sub-group	Rank	% of Niacin Contribution	Cumulative % of Niacin Contribution
**Main Group**			
bread, rolls, bread products	1	11.22	11.22
flour, bran, cooking ingredients	2	3.83	15.06
ready-to-eat cereal	3	1.50	16.55
rice, cooked grains	4	1.30	17.85
pizza, pasta and other flour dishes	5	1.29	19.14
**Sub-group**			
bread and rolls	1	9.62	9.62
wheat flour	2	3.79	13.42
quick breads and other bread products	3	1.60	15.01
breakfast cereals	4	1.50	16.51
pasta, macaroni, noodles	5	0.92	17.44
rice	6	0.78	18.21
groats and cereal grains	7	0.52	18.73
pizza and other flour dishes	8	0.37	19.10
other flours	9	0.04	19.14

Table S24. Food sources of vitamin B6 contribution from cereals and cereal products in the average Polish diet

Main Group<br>Sub-group	Rank	% of Vitamin B6 Contribution	Cumulative % of Vitamin B6Contribution
**Main Group**			
bread, rolls, bread products	1	8.43	8.43
flour, bran, cooking ingredients	2	2.47	10.90
rice, cooked grains	3	1.96	12.87
ready-to-eat cereal	4	1.56	14.42
pizza, pasta and other flour dishes	5	1.10	15.52
**Sub-group**			
bread and rolls	1	7.49	7.49
wheat flour	2	2.41	9.90
breakfast cereals	3	1.56	11.45
groats and cereal grains	4	1.04	12.49
quick breads and other bread products	5	0.95	13.44
rice	6	0.93	14.37
pasta, macaroni, noodles	7	0.83	15.20
pizza and other flour dishes	8	0.26	15.46
other flours	9	0.06	15.52

Table S25. Food sources of folate contribution from cereals and cereal products in the average Polish diet

Main Group<br>Sub-group	Rank	% of Folate Contribution	Cumulative % of Folate Contribution
**Main Group**			
bread, rolls, bread products	1	20.67	20.67
flour, bran, cooking ingredients	2	6.34	27.00
pizza, pasta and other flour dishes	3	3.27	30.28
ready-to-eat cereal	4	1.87	32.14
rice, cooked grains	5	1.43	33.57
**Sub-group**			
bread and rolls	1	16.96	16.96
wheat flour	2	6.27	23.24
quick breads and other bread products	3	3.70	26.94
pasta, macaroni, noodles	4	2.76	29.70
breakfast cereals	5	1.87	31.57
groats and cereal grains	6	0.73	32.30
rice	7	0.70	33.00
pizza and other flour dishes	8	0.51	33.51
other flours	9	0.07	33.57

Table S26. Food sources of vitamin B12 contribution from cereals and cereal products in the average Polish diet

Main Group<br>Sub-group	Rank	% of Vitamin B12 Contribution	Cumulative % of Vitamin B12 Contribution
**Main Group**			
bread, rolls, bread products	1	1.84	1.84
pizza, pasta and other flour dishes	2	0.83	2.67
flour, bran, cooking ingredients	3	0.55	3.21
ready-to-eat cereal	4	0.17	3.39
**Sub-group**			
quick breads and other bread products	1	1.17	1.17
bread and rolls	2	0.67	1.84
wheat flour	3	0.55	2.39
pasta, macaroni, noodles	4	0.45	2.84
pizza and other flour dishes	5	0.38	3.21
breakfast cereals	6	0.17	3.39

Table S27. Food sources of vitamin A contribution from cereals and cereal products in the average Polish diet

Main Group Sub-group	Rank	% of Vitamin A Contribution	Cumulative % of Vitamin A Contribution
**Food Group**			
bread, rolls, bread products	1	3.25	3.25
pizza, pasta and other flour dishes	2	1.41	4.66
ready-to-eat cereal	3	0.05	4.71
**Sub-group**			
quick breads and other bread products	1	2.38	2.38
pasta, macaroni, noodles	2	0.98	3.36
bread and rolls	3	0.88	4.24
pizza and other flour dishes	4	0.42	4.66
breakfast cereals	5	0.05	4.71

Table S28. Food sources of vitamin D contribution from cereals and cereal products in the average Polish diet

Main Group<br>Sub-group	Rank	% of Vitamin D Contribution	Cumulative % of Vitamin D Contribution
**Main Group**			
bread, rolls, bread products	1	4.61	4.61
pizza, pasta and other flour dishes	2	0.81	5.41
**Sub-group**			
quick breads, bread products	1	3.04	3.04
bread and rolls	2	1.56	4.61
pasta, macaroni, noodles	3	0.54	5.15
pizza and other flour dishes	4	0.27	5.41

Table S29. Food sources of vitamin E contribution from cereals and cereal products in the average Polish diet

Main Group<br>Sub-group	Rank	% of Vitamin E Contribution	Cumulative % of Vitamin E Contribution
**Main Group**			
bread, rolls, bread products	1	12.27	12.27
pizza, pasta and other flour dishes	2	1.02	13.29
flour, bran, cooking ingredients	3	0.98	14.28
ready-to-eat cereal	4	0.83	15.11
rice, cooked grains	5	0.39	15.50
**Sub-group**			
bread and rolls	1	8.37	8.37
quick breads and other bread products	2	3.90	12.27
wheat flour	3	0.97	13.24
breakfast cereals	4	0.83	14.06
pizza and other flour dishes	5	0.57	14.63
pasta, macaroni, noodles	6	0.46	15.09
rice	7	0.23	15.31
groats and cereal grains	8	0.16	15.48
other flours	9	0.02	15.50

Table S30. Food sources of vitamin C contribution from cereals and cereal products in the average Polish diet

Main Group<br>Sub-group	Rank	% of Vitamin C Contribution	Cumulative % of Vitamin C Contribution
**Main Group**			
pizza, pasta and other flour dishes	1	0.78	0.78
ready-to-eat cereal	2	0.53	1.31
bread, rolls, bread products	3	0.25	1.55
**Sub-group**			
pasta, macaroni, noodles	1	0.59	0.59
breakfast cereals	2	0.53	1.11
pizza and other flour dishes	3	0.19	1.31
quick breads and other bread products	4	0.15	1.45
bread and rolls	5	0.10	1.55
